# The therapeutic potential of targeting Oncostatin M and the interleukin-6 family in retinal diseases: A comprehensive review

**DOI:** 10.1515/biol-2022-1023

**Published:** 2024-12-31

**Authors:** Tommaso Mori, Nareshkumar Ragavachetty Nagaraj, Pier Luigi Surico, Wenjing Zhou, Uday Pratap Singh Parmar, Fabiana D’Esposito, Caterina Gagliano, Mutali Musa, Marco Zeppieri

**Affiliations:** Department of Pathology, University of California, San Diego, La Jolla, CA, 92093, United States of America; Department of Ophthalmology, Campus Bio-Medico University Hospital, Rome, 00128, Italy; Schepens Eye Research Institute of Mass Eye and Ear, Harvard Medical School, Boston, MA, 02114, United States of America; Department of Ophthalmology, Government Medical College and Hospital, Chandigarh, 160030, India; Imperial College Ophthalmic Research Group (ICORG) Unit, Imperial College, London, NW1 5QH, United Kingdom; Department of Medicine and Surgery, University of Enna “Kore”, Piazza dell’Università, 94100, Enna, EN, Italy; Eye Clinic Catania University San Marco Hospital, Viale Carlo Azeglio Ciampi, 95121, Catania, Italy; Department of Optometry, University of Benin, Benin City, 300238, Edo State, Nigeria; Department of Ophthalmology, University Hospital of Udine, p.le S. Maria della Misericordia 15, 33100, Udine, Italy

**Keywords:** Oncostatin M, retinal diseases, IL-6 family, cytokine modulation, therapeutic targets

## Abstract

Retinal diseases, which can lead to significant vision loss, are complex conditions involving various cellular and molecular mechanisms. The interleukin-6 (IL-6) family, particularly Oncostatin M (OSM), has garnered attention for their roles in retinal inflammation, angiogenesis, and neuroprotection. This comprehensive review explores the dual nature of OSM and other IL-6 family members in retinal pathophysiology, highlighting their contribution to both degenerative and regenerative processes. The review also examines current research on OSM’s interaction with key signaling pathways and discusses the potential of OSM and the IL-6 family as potential therapeutic targets. Understanding these mechanisms could lead to innovative treatments that modulate OSM activity, offering new avenues for managing retinal diseases and contributing to the development of more effective interventions.

## 1 Introduction

Retinal diseases such as age-related macular degeneration (AMD), diabetic retinopathy (DR), and uveitis are major causes of vision impairment and blindness worldwide [1]. These conditions involve complex processes such as chronic inflammation, oxidative stress, and abnormal angiogenesis. Inflammation, in particular, plays a crucial role in the progression of these diseases by disrupting the delicate balance of pro- and anti-inflammatory cytokines, thus leading to tissue damage and abnormal blood vessel growth [2]. Cytokines are small signaling proteins that regulate various aspects of the immune response, including inflammation, cell proliferation, and cell death. Among these, the interleukin-6 (IL-6) family of cytokines, including Oncostatin M (OSM), has received significant attention due to their dual role in both promoting and resolving inflammation. These cytokines are implicated in the development of various inflammatory and autoimmune diseases, including those affecting the retina [3].

This review aims to explore the potential therapeutic effects of OSM and the broader IL-6 family of cytokines in retinal diseases. By examining the molecular mechanisms through which these cytokines influence the health and disease of the retina, as well as by reviewing preclinical and clinical studies, this article aims to provide a comprehensive understanding of their potential as targets for novel therapies. Additionally, this review will discuss the challenges and future directions for translating these findings into clinical practice.

## 2 OSM and the IL-6 family

OSM is a significant member of the IL-6 cytokine family, characterized by the presence of the gp130 signal receptor subunit in their receptor complexes. This family includes various cytokines such as IL-6, IL-11, ciliary neurotrophic factor (CNTF), leukemia inhibitory factor (LIF), cardiotrophin-1 (CT-1), cardiotrophin-like cytokine factor 1, and IL-27 [4]. OSM was first discovered by Zarling et al. in the U-937 human lymphoma cell line [5]. The gene encoding human OSM is located on chromosome 22q12.2. The OSM gene encodes a polypeptide that initially consists of 252 amino acid residues. This polypeptide includes an N-terminal sequence of 25 amino acids and a C-terminal sequence of 32 amino acids. After proteolytic processing, the mature OSM protein consists of 195 amino acid residues. The mature human OSM protein has a molecular weight of approximately 28 kDa. Structurally, OSM is characterized by four α-helices arranged in an “up–up–down–down” topology [6–8]. This structural arrangement is typical of cytokines in the IL-6 family and is critical for their biological activity. OSM is widely expressed *in vivo* and plays a crucial role in various physiological and pathological processes. Several types of immune cells are known to produce and secrete OSM [9–12]. The receptor complexes for OSM are heterodimers, and their composition defines the type of signaling they mediate. Two main types of OSM receptor (OSMR) complexes exist: type I and type II [13,14]. The type I OSMR complex consists of the α subunit gp130 and the β subunit leukemia inhibitory factor receptor (LIFR) β subunit and the type II OSMR complex is composed of the α subunit gp130 and OSMR β subunit [15]. The receptor complex diversity mediates the different biological functions of OSM through the activation of multiple signaling pathways. The OSMR complex operates in a dynamic and species-specific manner, with its activation dependent on the presence of its subunits and ligand (OSM) [16]. In the absence of OSM, the two subunits of the OSMR complex, gp130 and either OSMR or LIFR, exist separately in a non-associated state. This separation prevents spontaneous activation of the downstream signaling pathways. When OSM is present, it first forms a low-affinity heterodimer with gp130. This initial interaction is crucial for the subsequent recruitment of the second subunit. After forming a heterodimer with gp130, the complex then recruits either the OSMR or LIFR subunit, depending on the specific receptor complex involved. The recruitment leads to the formation of a high-affinity receptor complex that is now capable of signaling [16]. Computational techniques including homology modeling, protein–protein docking, and molecular dynamics simulations were used by Du et al. to identify several critical amino acid residues involved in the binding process between OSM and OSMR [17]. These residues are crucial for the stability and specificity of the interaction, ensuring that OSM can effectively bind and activate its receptor, revealing potential “hot spots” that could serve as targets for inhibitor design to block this pathway. Moreover, it has been showed the OSM/OSMR signaling module exhibits a unique microenvironment-restricted expression pattern, suggesting that targeting this pathway could mitigate the side effects associated with anti-IL-6 therapies, strengthening the demonstrated safety and tolerability of humanized anti-OSM antibodies as a potential therapeutic strategy for inflammatory diseases and cancer treatment [18]. Also, it has been revealed that OSM and LIF share structural similarities, which explains why both cytokines can bind to the LIFR receptor [17]. Despite these similarities, OSM and LIF have distinct biological functions, attributed to differences in their overall structure and the specific receptor subunits they recruit. In mice, OSM predominantly binds to type II OSMR complexes, which include gp130 and OSMR. Mouse OSM generally does not activate LIFR, except at very high concentrations, where it can weakly stimulate LIFR. There is a single report that mouse-derived OSM can activate LIFR specifically in mouse osteoblasts, suggesting a degree of context-dependent flexibility [16,19,20]. OSM from rats and humans can bind to both type I (gp130 and LIFR) and type II (gp130 and OSMR) receptor complexes within their respective species. This dual-binding capacity allows OSM from these species to participate in a broader range of biological functions and signaling pathways compared to mouse OSM [21]. The interaction between OSM and its receptor complexes is a finely tuned process that depends on the presence of specific receptor subunits and the structural characteristics of OSM. The formation of the OSMR complex, particularly the role of OSMR in type II complexes, enables OSM to activate unique signaling pathways that differentiate its functions from other cytokines in the IL-6 family. The species-specific nature of OSM binding further underscores the complexity and specificity of its biological activities, making OSM a critical cytokine in both normal physiology and disease states

## 3 Role of OSM and IL-6 family in immune response, inflammation, angiogenesis, and cell survival

Abnormal levels of OSM are indeed linked to various inflammatory diseases. Compared to healthy controls, OSM levels are elevated in patients with pulmonary fibrosis [22]. It has been demonstrated that increased the expression of OSM and OSMR in many inflammatory bowel disease lesions [23]. Elevated levels of Oncostatin M receptor beta subunit (OSMRβ) in fibroblasts and dermal endothelial cells have been observed in patients with scleroderma, a chronic autoimmune disease characterized by fibrosis, vascular abnormalities, and immune system dysregulation [24]. Studies have indicated that cytokines such as OSM and interleukin-31 (IL-31) play a critical role in promoting itch and inflammation in this condition, particularly through their interaction with dermal cells expressing IL-31RA (IL-31 receptor A) and OSMRβ [25]. Elevated levels of OSM in gingival crevicular fluid have been observed in patients with periodontal disease, and research indicates that OSM concentrations increase in correlation with the severity of the disease, progressing from early-stage periodontal disease to chronic periodontitis [26–28]. In patients with coronavirus disease 2019 (COVID-19), OSM levels have been found to be elevated in peripheral blood plasma compared to healthy controls. These elevated levels of OSM are associated with the severity of the disease, suggesting that OSM plays a significant role in the immune response and inflammation characteristic of severe COVID-19 cases [29,30]. The role of OSM in promoting angiogenesis, particularly in the context of cancer, is supported by accumulating evidence [31]. OSM, a cytokine in the IL-6 family, can significantly influence angiogenesis through its effects on capillary endothelial cells and its activation of various signaling pathways. The recombinant human OSM has been shown to activate the STAT3 signaling pathway in endothelial cells. This activation leads to increased angiogenesis, particularly in tumor environments where new blood vessels are required to supply nutrients and oxygen to the growing tumor mass [32]. In osteosarcoma, a highly aggressive bone cancer, the activation of the OSM/JAK2/STAT3 axis has been linked to both increased angiogenesis and the invasive behavior of cancer cells. The upregulation of MMP2 and vascular endothelial growth factor (VEGF) by this pathway not only promotes the formation of new blood vessels but also enhances the cancer cell’s ability to invade surrounding tissues, contributing to the aggressive nature of osteosarcoma [33]. Neutrophils, which are often recruited to the tumor microenvironment, can produce OSM. Neutrophil-derived OSM interacts with the type II OSMR (composed of gp130 and OSMRβ) on breast cancer cells, leading to the induction of VEGF expression [34]. Indeed, while OSM and other IL-6 family cytokines share the ability to activate the STAT3 signaling pathway, their effects on angiogenesis can be inconsistent. This variability is influenced by several factors, including the specific cytokine involved, the cellular context, and the microenvironment. CNTF exhibits anti-angiogenic effects in vascular endothelial cells and the retina, suggesting that CNTF-induced STAT3 signaling can inhibit rather than promote angiogenesis in certain contexts. This contrasts with the generally pro-angiogenic effects of other IL-6 family cytokines and highlights the complexity of cytokine signaling pathways. This study sheds light on the nuanced role of STAT3 in angiogenesis and reveals the complex and context-dependent role of STAT3 signaling in angiogenesis, particularly in response to different IL-6 family cytokines like CNTF and OSM [35]. The findings underscore that the effects of STAT3 on angiogenesis are not uniform but are influenced by the specific cytokine involved, the concurrent activation of other signaling pathways, and the overall cellular environment. IL-6, a key cytokine in the IL-6 family, has been shown to induce the expression of VEGF mRNA in various cancer cell lines, including A431 cells (a human epidermoid carcinoma cell line) and C6 cells (a rat glioma cell line). This induction of VEGF expression highlights IL-6’s role in promoting angiogenesis, particularly in the context of tumor growth [36]. The study by Wei et al. using nude mice demonstrated that IL-6 plays a significant role in promoting tumor growth in human cervical cancer, specifically in the C33A cell line, through a mechanism that is dependent on VEGF-mediated angiogenesis [37]. In several cancers, including hepatocellular carcinoma, renal cell carcinoma, colorectal cancer, and glioblastoma, increased levels of circulating IL-6 have been linked to a poor response to therapies that target the VEGF/VEGFR pathway. These therapies include sunitinib, a tyrosine kinase inhibitor, and bevacizumab, an anti-VEGF antibody [36,38,39].

## 4 Molecular mechanisms and preclinical studies

Animal models have played an essential role in advancing our knowledge of retinal diseases and assessing potential treatments like OSM and the IL-6 family of cytokines. Among these models, rodents, particularly mice and rats, are favored due to their genetic adaptability and close resemblance to human retinal structures. One notable model is the oxygen-induced retinopathy (OIR) model in mice, which replicates the abnormal blood vessel growth seen in DR and ROP [40,41]. By subjecting mice to high oxygen levels followed by a return to normal levels, this model induces retinal ischemia and new blood vessel formation, mirroring the processes observed in human diseases. This model has been used to investigate the roles of various cytokines, including OSM and IL-6, in retinal inflammation and abnormal blood vessel growth and researchers have been trying to unveil the intricate and multifaceted roles of OSM and the IL-6 family in the context of retinal diseases. The IL-6 cytokine family is a diverse group of inflammatory and pleiotrophic cytokines, encompassing IL-27, IL-11, IL-6, LIF, CT-1, CLC, CNTF, and OSM. These cytokines utilize similar receptor complexes and commonly activate the STAT3 signaling pathway within cells. The angiomodulatory effect of CNTF-driven STAT3 signaling in different mouse models of vasoproliferative eye diseases involves both a direct anti-angiogenic impact on vascular endothelial cells and an indirect effect mediated by Müller cells [42,43]. Also, intravitreal injection of LIF can increase the vascular density in contrast to the OSM injection through changes in the cathepsin B and L expression [44]. OSM, in particular, exhibits a duality in its effects, displaying both protective and detrimental impacts contingent on the disease stage and the specific retinal microenvironment. Other preliminary studies showed that OSM treatment promotes the proliferation of choroidal and retinal endothelial cells, in contrast to its effect on aortic endothelial cells [45]. Rapp et al. observed a positive angiomodulatory effect of OSM following its intravitreal injection in the OIR model. The treatment activated STAT3 signaling in various retinal cell types, including Müller and vascular endothelial cells. *In vitro* studies indicate that OSM influenced the secretory profile of Müller cells. OSM promotes the regeneration of rods and cone photoreceptors in the early stage of degeneration via interacting with muller cells through activating STAT3. Also, it has been the regenerative role of OSM reported in the muscle, bone [46], and heart [47]. Additionally, RNA sequencing of isolated retinal vascular endothelial cells from OIR P17 mice revealed a transcriptomic shift resulting from OSM treatment at P12. These findings highlight the critical role of Müller cells in vasoproliferative eye diseases and suggest that the effects of angiogenic agents may vary significantly between *in vitro* and *in vivo* environments. Also, it suggests that angiomodulatory agents like OSM might produce unanticipated and occasionally opposing effects *in vivo* versus *in vitro*, depending on the cell types involved within the intricate retinal environment. OSM shows a positive angiomodulatory influence on retinal angiogenesis, with Müller cells being key contributors to vascular homeostasis. Another study highlights the intricate role of STAT3 signaling in vascular endothelial cells in response to cytokine treatment, leading to diverse angiogenic outcomes [47]. The angiomodulatory effects of STAT3 appear to be influenced by the activity of other intracellular signaling pathways and its specific localization within the cytosol, mitochondria, or nucleus. OSM and CNTF differ in their intracellular signaling mechanisms and STAT3 specificity, resulting in distinct transcriptomic profiles and metabolic activities [35].

Another significant model is the streptozotocin (STZ)-induced diabetic mouse model, which allows for the study of the long-term impacts of high blood sugar on the retina, particularly in the context of DR. Through STZ administration, the destruction of pancreatic β cells occurs, leading to elevated blood sugar levels and consequent damage to retinal blood vessels [48]. Robinson et al. demonstrated that inhibiting IL-6 trans-signaling significantly reduces diabetes-induced oxidative damage systemically and in the retina in an STZ-induced diabetic mouse model [49]. Another study discovered that high levels of IL-6, a proinflammatory cytokine in the eye, were significantly linked to the advancement of proliferative diabetic retinopathy (PDR) and poor outcomes after eye surgery. This indicates that IL-6 within the eye may play a crucial role in the development of abnormal blood vessel growth associated with PDR, possibly by increasing VEGF expression [50]. Mason et al. systematically review and analyze the changes in cytokine levels in the eyes of patients with nonproliferative DR. The meta-analysis highlights significant alterations in inflammatory cytokines, which could potentially contribute to the pathophysiology of the disease and may inform future therapeutic strategies [51]. Moreover, other constituents of the IL-6 family, such as LIF and CNTF, have exhibited potential as neuroprotective agents in diverse models of retinal degeneration. For instance, LIF has demonstrated the ability to shield and protect the choriocapillaris, and yet, the photoreceptors, in a mouse model of dry-AMD, induced with sodium iodate [52]. Yang et al. showed that a single intravitreal injection of CNTF-NP or OSM-NP provided protection for retinal ganglion cells in an acute glaucoma model and safeguarded photoreceptors and vision for over 70 days in a rat model of retinitis pigmentosa [53].

## 5 Clinical studies and trials

The investigation of OSM and the IL-6 family as potential therapeutic targets in retinal diseases has progressed from animal experiments to clinical trials, summarized in Table 1. Clinical studies have mainly focused on the role of IL-6 in retinal diseases. One important clinical trial examined the use of tocilizumab, a monoclonal antibody targeting IL-6, in patients with macular edema related to non-infectious uveitis. The trial demonstrated that tocilizumab (at both 4 and 8 mg/kg doses) effectively enhances visual acuity, reducing vitreous haze and central macular thickness in eyes with noninfectious uveitis. Tocilizumab is generally well-tolerated but requires careful patient monitoring due to risks such as neutropenia and gastrointestinal perforations. It offers a new avenue for managing challenging cases of retinal inflammatory diseases [54].

**Table 1:** Anti-IL-6 and anti-OSM therapeutics in development and current indications

Molecule	Company/university	Structure	Target	Current indication (FDA approved)	Potential future application	Reference
Tocilizumab	Genentech/Roche	Monoclonal antibody	IL-6	Rheumatoid arthritis	Retinal inflammatory diseases, particularly non-infectious uveitis	[54]
Sarilumab	Regeneron/Sanofi	Fully human monoclonal antibody	IL-6Rα	Rheumatoid arthritis	Posterior segment non-infectious uveitis	[55]
EBI-031	Roche/Eleven Biotherapeutics	Monoclonal antibody	IL-6, IL-6R Complex	None	Diabetic macular edema	[56]
KSI-501	Kodiak Sciences	Bispecific inhibitor	IL-6, VEGF	None	Neovascular AMD, diabetic macular edema	[57]
GSK315234	GlaxoSmithKline	Humanized monoclonal antibody	OSM	None (Ph II discontinued)	Rheumatoid arthritis	[58]
GSK2330811	GlaxoSmithKline	Monoclonal antibody	OSM	None (Ph II discontinued)	Systemic scleroderma	[59]
WO2020127884A1	Université de Poitiers	Specific binding protein	OSM	None	Inflammatory skin diseases, cancer	[60]
US9550828B2	Boise State University	Small molecule inhibitor	OSM	None	Tumor cell detachment, invasion, metastasis	[61]

Sarilumab is a fully human anti-IL-6Rα mAb that blocks the IL-6 signaling pathways by binding to membrane and soluble forms of IL-6Rα. After receiving approval for rheumatoid arthritis, it is undergoing clinical trials for use in the management of posterior segment non-infectious uveitis. In the SATURN study, patients with non-infectious uveitis showed reduced vitreous haze and lower steroid dosing with 200 mg subcutaneous dose every 2 weeks. The treatment improved visual acuity and central macular thickness with fewer side effects than other therapies, but adverse events like neutropenia and elevated alanine aminotransferase levels were reported [55].

Designed specifically for intravitreal delivery, EBI-031 blocks both free IL-6 and the IL-6 receptor complex. Clinical trials have been conducted to assess the effectiveness and safety of EBI-031 in patients with diabetic macular edema [56].

The current Phase I clinical trial involving KSI-501 marks a significant leap forward in the medical field. This trial aims to combat neovascular AMD and Diabetic Macular Edema (DME) by targeting both IL-6 and VEGF. KSI-501 is an innovative bispecific inhibitor specifically engineered to concurrently address IL-6 and VEGF, with the goal of overcoming the limitations encountered in prior studies by combining anti-inflammatory and anti-angiogenic effects. Initial findings from this trial have displayed encouraging results in terms of safety and preliminary effectiveness, and more comprehensive data are anticipated to unveil the therapeutic potential of this dual-target approach [57].

Many trials are currently underway to evaluate the effectiveness of blocking the activation of OSM pathways, but none are focused on the retinal field. For instance, a humanized anti-OSM monoclonal antibody developed by GSK for rheumatoid arthritis (GSK315234) showed limited efficacy in clinical trials due to poor binding affinity and was eventually discontinued [58]. Another GSK anti-OSM monoclonal antibody (GSK2330811) demonstrated safety in early trials for systemic scleroderma but showed no significant efficacy, and all patients in the highest dose group experienced adverse effects [59]. Researchers have developed a specific binding protein targeting OSM to inhibit its interaction with gp130 or LIFR (WO2020127884A1), potentially applicable to inflammatory skin diseases and cancer [60]. Boise State University developed small molecule inhibitors targeting Site III of OSM (US9550828B2), particularly SMI-10B, to reduce tumor cell detachment and metastasis, confirmed through molecular dynamics simulation [61].

Upcoming clinical trials will explore the combination of cytokine modulation with established treatments, such as anti-VEGF therapies, to enhance therapeutic results. Moreover, there is growing interest in personalized medicine strategies, where patient-specific cytokine profiles could guide the selection and combination of therapies to optimize treatment effectiveness and minimize adverse effects. The potential for therapies targeting OSM in retinal diseases also continues to be an area of intense study, with preclinical data suggesting the potential neuroprotective benefits of OSM modulation in certain retinal conditions.

## 6 Therapeutic strategies and future perspectives

Modulating the activity of OSM and the IL-6 family offers a promising approach to treating retinal diseases. The cytokine network in the retina is complex, and strategies to modulate this network must be carefully designed to achieve therapeutic benefits without unintended adverse effects. One approach to cytokine modulation involves using inhibitors targeting the IL-6 receptor (e.g., tocilizumab) are being assessed for their ability to halt disease progression by blocking IL-6 signaling pathways [62]. These therapies aim to reduce the inflammatory and angiogenic processes that contribute to retinal degeneration. Another potential strategy involves using agonists that activate protective cytokine pathways. For instance, recombinant forms of neuroprotective cytokines like CNTF have been investigated for their ability to slow down the degeneration of light-sensitive cells in conditions like retinitis pigmentosa [53]. CNTF works by activating survival pathways that counteract signals that lead to cell death in the retina, thereby preserving vision. Despite promising preclinical results, challenges exist in ensuring the effective and targeted delivery of such therapies to the retina. One of the key challenges in cytokine modulation is achieving precise control over cytokine activity. Over-inhibition of cytokines like IL-6 could impair the retina’s natural defense mechanisms, leading to unintended consequences. Conversely, excessive activation of protective cytokines could potentially trigger unintended effects or lead to immune system imbalances. Therefore, the development of cytokine-based therapies must take into account the balance between efficacy and safety [63].

Combination therapies that target multiple pathways simultaneously represent a promising approach to enhancing treatment efficacy in retinal diseases. For example, an ongoing Phase I clinical trial is investigating a bispecific inhibitor that targets both IL-6 and VEGF [57]. By simultaneously addressing the inflammatory and angiogenic components of retinal diseases, this dual-target approach has the potential to provide superior therapeutic outcomes compared to monotherapy. Additionally, combining cytokine modulation with existing treatments, such as anti-VEGF therapies, may offer synergistic effects. For instance, in AMD and DME, where VEGF plays a central role in pathological angiogenesis, adding cytokine-targeting agents could further suppress disease progression by modulating the inflammatory environment that supports neovascularization. Personalized medicine also holds great potential in the context of cytokine modulation for retinal diseases. By tailoring treatments based on individual patient profiles, including specific cytokine expression patterns and genetic predispositions, clinicians could optimize therapeutic efficacy while minimizing side effects. For example, patients with elevated levels of IL-6 or OSM may benefit more from therapies targeting these cytokines, while others may require different approaches. Future research will likely focus on identifying biomarkers that can guide personalized treatment strategies and on developing advanced delivery systems that ensure precise targeting of cytokine modulators to the retina. As our understanding of the cytokine network in retinal diseases deepens, the potential for personalized and combination therapies to transform the management of these conditions will continue to grow.

## 7 Conclusion

In this review, we have examined the various roles of OSM and the IL-6 family of cytokines in relation to retinal diseases. The evidence emphasizes the significant influence of these cytokines on inflammation, neovascularization, and cell survival within the retina. Preclinical studies show the potential effectiveness of targeting these pathways while emerging clinical data indicate encouraging avenues for therapeutic intervention. The increased expression of IL-6 and OSM in retinal diseases, such as DR and AMD, may reflect a failure of anti-inflammatory pathways to properly regulate chronic low-grade inflammation, which is a key feature in the progression of these diseases. This disruption could lead to a compensatory yet maladaptive overproduction of pro-inflammatory cytokines, contributing to ongoing retinal damage. More studies need to determine whether this cytokine upregulation is a primary mechanism or a secondary response to deficiencies in anti-inflammatory signaling, which could offer new insights into therapeutic approaches targeting inflammation regulation in retinal diseases.

**Figure 1:**
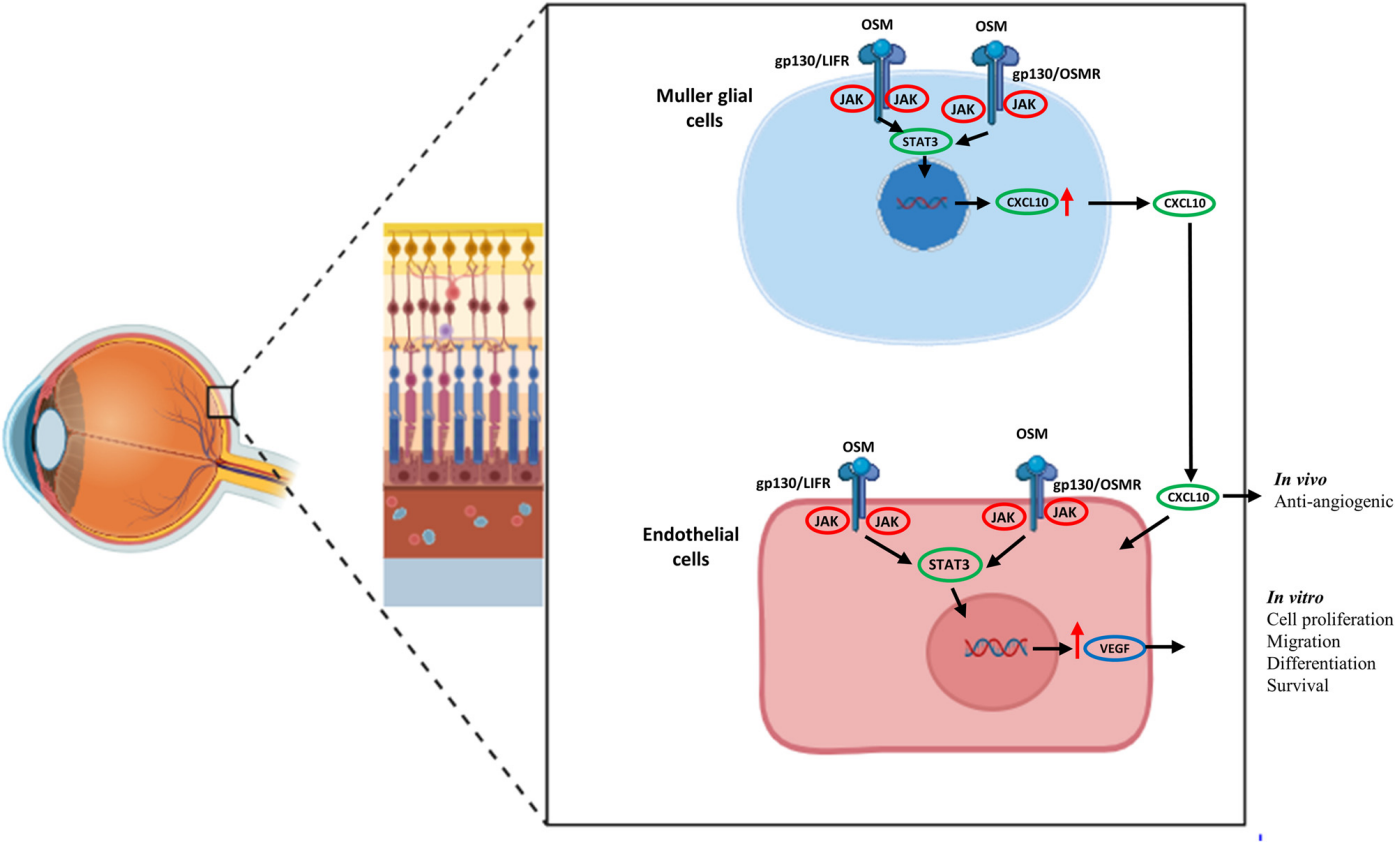
Graphical representation of signaling pathways influenced by OSM in retinal cells. Angiomodulatory effect of OSM in endothelial cells (*in vitro*) via mullers cells through stat3 activation (*in vivo*) and thereby increasing CXCL 10 can lead to anti-angiogenic in the retinal vasculature.

To address both inflammation and vascular remodeling, targeting the OSM pathways may offer a promising alternative to current therapies for retinal diseases. While potential challenges like antibody formation exist, strategies to minimize immunogenicity and combine these treatments with anti-VEGF agents may enhance therapeutic efficacy. The optimal treatment regimen will depend on patient-specific responses, and further studies are needed to refine these approaches and ensure long-term effectiveness. Moreover, there are challenges, such as the requirement for specific and safe delivery methods and the management of potential side effects. Future research should concentrate on refining these therapeutic approaches, including exploring combination therapies and personalized medicine strategies tailored to individual patient profiles. Continued clinical investigation is essential to fully realize the potential of OSM and IL-6 family cytokine modulation, which could revolutionize therapeutic approaches for retinal diseases and enhance patient outcomes (Figure 1).

Table 1 summarizes various anti-IL-6, anti-OSM, and therapeutic agents under investigation or clinical use, highlighting their molecular structure, targeted pathways, current FDA-approved indications, and potential future applications.
